# The Burden of Survivorship: Survivor Guilt and Its Association with Psychiatric Sequelae in COVID-19 Patients

**DOI:** 10.3390/jcm12093117

**Published:** 2023-04-25

**Authors:** Mariagrazia Palladini, Mario Gennaro Mazza, Andrea Scalabrini, Patrizia Rovere Querini, Sara Poletti, Francesco Benedetti

**Affiliations:** 1 Psychiatry & Clinical Psychobiology, Division of Neuroscience, IRCCS Scientific Institute Ospedale San Raffaele, 20127 Milan, Italy; 2 PhD Program in Cognitive Neuroscience, Vita-Salute San Raffaele University, 20132 Milan, Italy; 3 Vita-Salute San Raffaele University, 20132 Milan, Italy; 4 Department of Human and Social Sciences, University of Bergamo, 24129 Bergamo, Italy; 5 Division of Immunology, Transplantation and Infectious Diseases, IRCCS San Raffaele Scientific Institute, 20132 Milan, Italy

**Keywords:** COVID-19, survivor guilt, post-traumatic distress, depression, depressive cognitive style

## Abstract

COVID-19 survivors struggle with intense depressive and post-traumatic symptoms in sub-acute stages. Survivor guilt may affect post-acute psychopathology. Herein, we aim to unveil the potential affective mechanism underpinning post-COVID psychiatric implications by focusing on the association of survivor guilt with psychopathology and maladaptive attributional style. At one month after discharge, we evaluated symptoms of depression on The Zung Severity Rating Scale (ZSDS), post-traumatic distress on Impact of Event Scale-Revised (IES-R), and sleep disturbances on the Women’s Health Initiative Insomnia Rating Scale (WHIIRS) in 195 COVID-19 survivors. Interpersonal Guilt Rating Scale (IGRS-15) rated survivor guilt. A discrepancy score between the burden of depression and post-traumatic distress symptoms was computed individually. Dysfunctional depressive attributions were assessed through the Cognition Questionnaire (CQ). Survivor guilt significantly predicts all evaluated psychopathological dimensions. Moreover, higher rates of survivor guilt were associated with an overlap between post-traumatic and depressive symptomatology, thus suggesting that survivor guilt equally sustains both psychiatric manifestations. Finally, survivor guilt fully mediated the relationship between dysfunctional depressive attributions and the discrepancy index. Our results confirm survivor guilt as a clinically relevant form of suffering related to psychopathological dimensions of post COVID-19 infection, gaining the status of a specific phenomenon and a promising treatment target.

## 1. Introduction

As the coronavirus disease (COVID-19) spread around the world, accumulating evidence suggested that COVID-19 survivors are at an increased risk of psychiatric outcomes after the infection [1,2,3]. It is now clear that the burden of the disease extends beyond acute and post-infection organic complications, triggering prolonged psychopathological symptomatology and, therefore, posing an outstanding challenge to mental health care systems. Depressive and post-traumatic symptoms are regularly documented in recent literature, representing a common psychopathological pattern acknowledged in COVID-19 survivors both during and after hospitalization [4], with meta-analytical evidence in large samples affirming a pooled prevalence of depression of 31% [5].

Considering the heterogeneity of clinical manifestations of depression, whose phenotypes frequently entails either ‘hot’ or ‘cold’ cognitive dysfunctions as seen also in depressed COVID-19 survivors [6,7], mental health professionals warned about the possibility of irrational survivor guilt feelings in the aftermath of COVID-19 [8], and searching for specific targets for psychotherapeutic interventions. Survivor guilt is usually regarded as an unpleasant feeling originating from the belief that achieving a greater degree of health and well-being in life may occur to the disadvantage of someone else [9]. Traumatic occurrences may precipitate these feelings, inducing an exaggerated sense of responsibility for others [10]. According to this interpersonal view [11], dysfunctional attributional styles in interpreting life events represent the ideal background to exacerbate survivor guilt that, which, in turn, fosters psychopathology [12,13,14]. In this regard, a higher proneness to both depressive and post-traumatic manifestations is well-documented in those who experienced strong survivor guilt feelings [15,16,17], especially when linked to maladaptive attributional attitudes [18]. For its part, a global tendency to read events in a distorted, pessimistic way figures prominently in the development, maintenance, and prognosis of depression [19] and receives greater emphasis in the context of post-traumatic stress disorder (PTSD) as well [20]. Additionally, novel investigations suggest that a significant proportion of COVID-19 survivors who fell in the pathological range for depression also screens positive for post-traumatic distress [1,21,22,23], resulting in a more complex and heterogeneous psychopathological profile with a critical impact on treatment efficacy [24,25,26,27].

In the context of the COVID-19 pandemic, survivor may experience oppressive feelings of guilt arising from the belief of having infected family members or be fostered by questioning why they were spared while others succumbed to the novel disease [28]. Since the early phases of COVID-19 outbreak, experts argued that the pandemic can be regarded as a full-fledged traumatic event, forcing those infected with the virus to deal with their own and maybe loved ones’ uncertain prognosis or, at best, exposing them to a state of quarantine and isolation [29]. Taken together, this constellation of factors affects the susceptibility of COVID-19 survivors to post-traumatic and depressive symptomatology at short- and long-term [30], whose coexistence in the post-COVID-19 stages has been recently linked to interpersonal difficulties [22].

Notwithstanding the possible relevance of survivor guilt in affecting post-acute COVID-19 psychopathology, no study has investigated it in COVID-19 survivors. The purpose of the current study is to provide new insights into the affective mechanism underpinning psychiatric implications (i.e., depression, post-traumatic distress, and insomnia) in the post-COVID period, by focusing on the association of survivor guilt with psychopathology and maladaptive attributional style. In keeping with the above referenced literature, it seems plausible to assume survivor guilt as a major driver for co-occurring post-traumatic and depressive symptomatology.

In detail, our aims are (I) to explore the predictors of survivor guilt and its association with the psychiatric sequelae (i.e., depression, post-traumatic distress, insomnia) largely reported in COVID-19 survivors; (II) to investigate how survivor guilt is related to post-traumatic and depressive outcomes. More specifically, we aim to define to what extent survivor guilt represents a common factor for both outcomes or, alternatively, whether it plays a specific role in each psychopathological dimension. In a subsample of patients, we also aim to investigate the relationship between dysfunctional depressive attributional style, survivor guilt, and psychopathological dimensions.

## 2. Methods

### 2.1. Participants and Procedure

We evaluated 195 COVID-19 survivors during an ongoing prospective study carried out at the San Raffaele Hospital in Milan. Patients’ COVID-19 infection was assessed through radiological and clinical findings at the Emergency Department and confirmed by a positive real-time reverse-transcriptase polymerase chain reaction from a nasopharyngeal and/or throat swab. To keep a naturalistic study design, exclusion criteria were limited to age below 18 years and non-Italian speakers.

### 2.2. Measures

In addition to demographic and clinical data, including the family’s experience of illness (“Nobody else infected”, “somebody infected but not hospitalized”, “Somebody infected and hospitalized”) and setting of care (i.e., hospitalization, non-invasive ventilation, and intensive care unit admission), participants were requested to complete self-rated questionnaires aimed at assessing psychopathological status at one-month follow-up (35.82 ± 12.94 days after hospital discharge). The severity of post-acute COVID psychopathology was self-rated using the Zung Severity Depression Scale (ZSDS) [31] to investigate depressive symptomatology, the Impact of Event Scale Revised (IES-R) [32] to evaluate post-traumatic distress, and the Women’s Health Initiative Insomnia Rating Scale (WHIIRS) [33,34] to assess sleep disturbances, which all proved to be sensitive to COVID-19 triggered symptoms [1,2,35,36,37]. Previously validated cut-off scores were considered to determine the presence of psychopathology (ZSDS index ≥ 50; IES-R ≥ 33; WHIIRS ≥ 9).

To obtain a meaningful measure capable of gauging divergence in the individual burden of post-traumatic distress and depression, we calculated an IES-ZSDS discrepancy score index: (IES-R total score)/(88, maximum score) × 100 − (ZSDS score)/(100, maximum score) × 100 [38,39].

For the specific assessment of survivor guilt, referring to the distress people may experience when they assume good things happened to them were obtained at the expense of harming others (e.g., “I feel uncomfortable feeling better off than other people”), we administered The Interpersonal Guilt Questionnaire-Revised (IGRS-15) [9,40]. The inventory consists of 15 items regarding four kinds of irrational guilt feelings, which can arise from traumatic circumstances.

Moreover, to further investigate the cognitive-affective mechanism underpinning psychiatric outcomes, a sub-sample of 67 COVID-19 survivors was administered the Cognition Questionnaire (CQ) [41] to specifically evaluate negative thinking styles. The CQ provides a global measure of depressive cognitive style, which assesses dimensions of negative thinking in relation to several hypothetical events. The depressive attributional attitude is evaluated in respect to several positive, negative, or neutral brief scenarios, each with its own four fixed range of alternatives specifying different cognitive explanations.

### 2.3. Ethics

The authors assert that all procedures contributing to this work comply with the ethical standards of the relevant national and institutional committees on human experimentation and with the Helsinki Declaration of 1975, as revised in 2008. Informed consent was obtained from all subjects involved in the study. All procedures involving human patients were approved by the Ethics Committee of San Raffaele Hospital (COVID-BioB protocol NCT04318366).

### 2.4. Data Analysis

All the analyses were performed using a commercially available software package (StatSoft Statistica 12, Tulsa, OK, USA) and following standard computational procedures [42,43].

First, we explored the effect of demographics, setting of care, along with familiar experience of illness on survivor guilt (IGRS-15) through an analysis of variance (ANCOVA). Secondly, we investigated the role of survivor guilt in predicting psychological sequelae as rated on self-report measures at one month after hospital discharge, also accounting for age and sex. For this purpose, a GLM multivariate analysis (MANCOVA) was conducted, thus allowing to account for relationships between psychopathological measures entered as dependent variables. The effect of predictors was modelled in the context of General Linear Model, and statistical significance of the effect was computed by parametric estimates of predictor variables (least squares method).

Furthermore, to explore the effect of survivor guilt on IES-ZSDS discrepancy score, while considering the possible interaction between other independent variables such as age and sex, we implemented a Generalized linear model (GLMZ) with a homogeneity of slope design and an identity link function [44]. Parameter estimates were obtained using iterative re-weighted least squares maximum likelihood procedures. To infer the significance of the effect, the likelihood ratio statistic (LR) was reported, which provides a test of the increment in the log-likelihood of the model attributable to the respective current effect, thus yielding a measure of the incremental Chi-squared statistic associated with each effect.

Based on both the obtained results and existing literature, we explored the possible mediating role of survivor guilt in the association of maladaptive attributional style (CQ) and the IES-ZSDS discrepancy score measure, entering age and sex as nuisance covariates. Preliminary partial correlations of CQ with survivor guilt and the IES-ZSDS index were conducted [45]. Mediation analyses were performed using a non-parametric resampling bootstrapping technique [46] in an SPSS macro [47]. In the current study, 5.000 bootstraps resampling procedures were used to generate 95% confidence intervals for the indirect effect. The R-squared was also obtained as a goodness-of-fit measure for the mediation model.

The authors declare that all procedures contributing to this work comply with the ethical standards of the relevant national and institutional committees on human experimentation and with the Helsinki Declaration of 1975, as revised in 2008. Written informed consent to participate in the study was obtained from all participants. All procedures involving human patients were approved by the Ethics Committee of San Raffaele Hospital (COVID-BioB protocol NCT04318366).

## 3. Results

Demographic and clinical characteristics of COVID-19 survivors are summarized in Table 1.

The whole cohort included 63 females and 132 males (mean age 57.29 ± 10.41), all of whom were hospitalized. Among them, 147 (75.38%) received inpatients standard care, 34 (17.44%) required non-invasive ventilation support (i.e., CPAP), and 14 (7.18%) were admitted to intensive care unit. With regards to familiar experience of illness, 50 (25.64%) declared to be the only family member who contracted the virus, 109 (55.90%) affirmed to have at least one family member infected but managed at home, and 36 (18.46%) asserted that one or more family members were infected and needed hospital care. Considering specifically post-traumatic and depressive symptoms severity, 62/195 (31.8%) of patients scored in the pathological range in at least one of the two psychopathological dimensions. Among them, 31/62 (50%) screened positive for both, revealing a high prevalence of posttraumatic stress and depression co-occurrence in our sample.

Among demographic factors, only age was positively associated with the severity of survivor guilt (older age, worse guilt; β = 0.27, *F* = 14.57, *p* < 0.001). Setting of care and familiar experience of illness did not show significant effects.

A GLM multivariate analysis of the effect of survivor guilt on current psychopathological status (post-traumatic distress, depression, and insomnia) showed a multivariate significant effect of survivor guilt (Wilks *λ* = 0.81, *F* = 14.46, *p* < 0.001). Univariate testing significantly associated survivor guilt with all the psychopathological dimensions considered (IES-R: β = 0.43, *F* = 42.09, *p* < 0.001; ZUNG-index: β = 0.21, *F* = 9.86, *p* = 0.002; WHIIRS: β = 0.17, *F* = 5.43, *p* = 0.021), with the maximal effect size on post-traumatic distress (IES-R: *ƞ_p_*^2^ = 0.18; ZUNG-index: *ƞ_p_*^2^ = 0.05; WHIIRS: *ƞ_p_*^2^ = 0.03).

**Figure 1:**
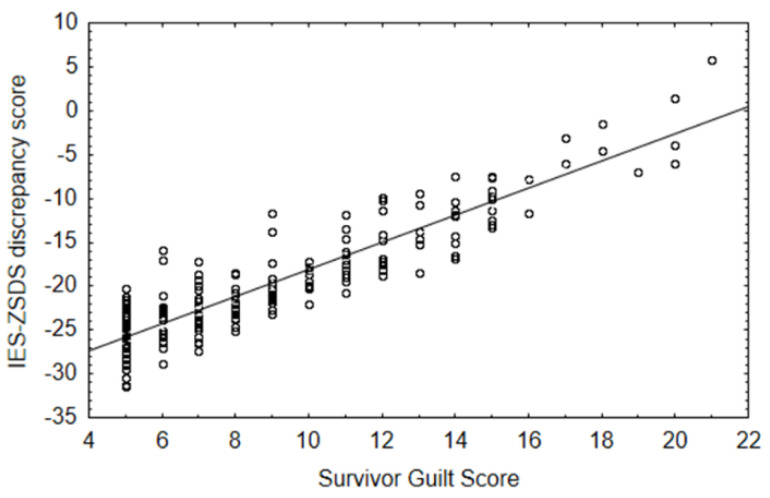
Effect of survivor guilt in predicting IES-ZSDS discrepancy score.

A GLZM homogeneity of slope analysis exploring the effect of survivor guilt on IES-ZSDS discrepancy, also considering age and sex as nuisance covariates, showed that survivor guilt significantly increases the log-likelihood of the model, being a reliable predictor of the discrepancy between IES and ZSDS index scores (*LR χ*^2^ = 31.12, *p* < 0.001). Inspection of parameters demonstrated a positive association between the predictor and the outcome (β = 1.75, Wald = 33.74, *p* < 0.001): an increase in survivor guilt is associated with higher values of IES-ZSDS index scores, where a higher level of this variable indicates prevalent post-traumatic symptomatology. However, inspection of the data (See Figure 1) shows that the maximum of the index reached in the sample is 0, meaning that the severity of post-traumatic and depressive symptomatology is similar in patients who also show high levels of survivor guilt, while depression predominates over post-traumatic distress when survivor guilt is low.

**Figure 2:**
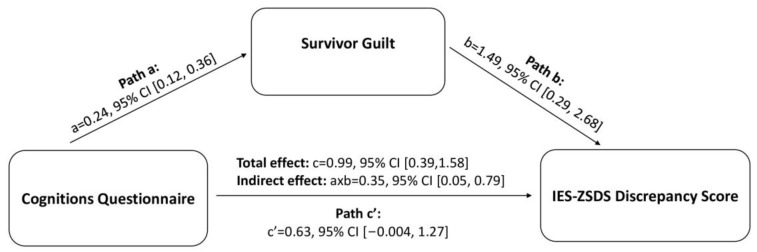
Mediation model of the effect of dysfunctional attributional style on IES-ZSDS discrepancy score. c’ = direct effect; c = total effect; 95 % Confidence Intervals (CI) are reported to evaluate the significance of the effects (a, b, axb, c’, c).

We then tested the effect of survivor guilt in mediating the relationship between cognitive distortion (CQ score) and IES-ZSDS discrepancy, while also considering age and sex as nuisance covariates. Baron-Kenny assumptions for mediation modelling were met [45]: CQ was positively associated with both survivor guilt (*r* = 0.40, *p* = 0.001) and IES-ZSDS (*r* = 0.38, *p* = 0.001), while also controlling for the third element (survivor guilt: *rp* = 0.31, *p* = 0.011; IES-ZSDS: *rp* = 0.28, *p* = 0.023). The mediation model revealed a significant indirect effect (axb = 0.35, 95% CI = 0.051, 0.79), thus confirming the mediating role of survivor guilt in the relationship between dysfunctional attributional style and depressive/post-traumatic symptoms (See Figure 2).

The total effect of CQ on IES-ZSDS scores was significant (c = 0.99, *t* = 3.32, *p* = 0.002). The direct effect was not significant (c’ = 0.63, *t* = 1.99, 95% CI = −0.004, 1.27), suggesting a full mediation effect. Analyses also confirmed the effect of CQ scores on the mediator (survivor guilt; a = 0.24, 95% CI = 0.12, 0.36), and the effect of the mediator on the outcome variable (b = 1.49, 95% CI = 0.29, 2.68), controlling for CQ scores. The model achieved high performance for the combined effect of CQ and survivor guilt scores on the IES-ZSDS discrepancy index (*R*^2^ = 0.47, *F* = 4.56, *p* = 0.002).

## 4. Discussion

This is the first study to investigate the role of survivor guilt in determining psychiatric outcomes of COVID-19, focusing on survivor guilt as a potential affective pathway resulting in post-traumatic and depressive manifestations in COVID-19 survivors. Our results highlighted (i) a positive association between age and survivor guilt; (ii) worsening of the severity of depression, post-traumatic, and sleep disturbances with higher survivor guilt; (iii) an increase in survivor guilt corresponds to an overlap of post-traumatic and depressive frames; (iv) secondarily, survivor guilt fully mediates the relationship between dysfunctional cognitive attributions and the divergence in post-traumatic and depressive symptoms severity, as rated on IES-ZSDS discrepancy index. Together, our findings show the key role of survivor guilt as a common affective ground underlying post-traumatic and depressive-like symptomatology.

The main finding of our study showed that high levels of survivor guilt correspond to a flattening of the distance between post-traumatic and depressive severity as represented by the IES-ZSDS discrepancy score, suggesting that survivor guilt equally contributes to both forms of psychopathology, probably identifying a common ground of the two. Indeed, novel literature stresses the importance of survivor guilt as a distinct psychological issue, which can be identified or not in both PTSD and Major Depressive Disorder (MDD) [12]. This is critical, especially considering the impressive rates of clinically relevant depressive and post-traumatic symptoms experienced by COVID-19 survivors [48], with a large co-occurrence of the two [49].

Intriguingly, we found a positive relationship between age and survivor guilt, without any other effects of demographic and clinical features. The only few studies available on survivor guilt [50,51] applied to individuals diagnosed with serious threatening illnesses provide support for marked guilt emotions in case a survivor compares themselves with younger people left behind. Therefore, we speculate that those who are most vulnerable may feel particularly guilty for having escaped a tragic ending while someone else with higher chances of surviving failed. Otherwise, considering age as a major risk factor for mortality among those who contract COVID-19 [52], one can hypothesize that elderly patients are more likely to have experienced intense survivor guilt feelings in relation to comparable individuals who finally died. Additionally, it is noteworthy that there was no association between any of the clinical features related to the severity of infection (e.g., setting of care). This is in line with recent insights suggesting that survivor guilt is linked with psychosocial constraints induced by the COVID-19 outbreak, such as limitations of visits to hospitalized loved ones, lack of information about their physical conditions, and consequently, an impossibility to prepare for their loss [53].

We also demonstrated that survivor guilt predicts both post-traumatic and depressive manifestations, being also related to insomnia. Notably, survivor guilt exerted a major effect on PTSD. Irrational survivor guilt represents a crucial component in post-traumatic stress disorder, being associated with more severe forms of PTSD [54] and acting as a maintenance factor [55], also inducing substance abuse [56] and boosting suicide risk [57]. Despite experts tend to reference survivor guilt merely in relation to post-traumatic frame, it is involved in the onset of depression [17,58]. Adverse outcomes of survivor guilt implicate also sleep disturbances [59], especially when emerging within the context of post-traumatic distress [12,56].

Secondarily, we also pointed out that survivor guilt acts as a mediator in the association between dysfunctional attributional style and the burden of post-traumatic and depressive outcomes jointly considered. This finding further supports the role of survivor guilt as a core affective ground in explaining the association between maladaptive attributional styles and PTSD and depressive outcomes and complements previous insights. Available evidence supports dysfunctional attributional style as an antecedent [60] and regular feature [61] of depression, being particularly noticeable in the chronic form of the disorder and thus becoming a primary target of antidepressant treatment [62]. Likewise, dysfunctional attributional style is widely recognized as a risk factor for PTSD, playing a key role in the maintenance of symptomatology [20,63,64]. Furthermore, research indicates pessimistically tuned attributional attitude as a shared underpinning of comorbid depression and PTSD [65,66], as well as a candidate to explain the impressive rate of overlap between the two. Herein, our results shed new light on that pathway, revealing a fully mediating effect of survivor guilt in the context of post-COVID psychiatric sequelae. As such, survivor guilt may become a helpful lens through which clinicians can frame depressive and post-traumatic manifestation resulting from COVID-19 infection, with potential usefulness for mental health services, especially considering the widespread demand for clinical interventions in the aftermath of COVID-19 and the necessity of targeting specific brief psychotherapeutic interventions [67,68].

The COVID-19 outbreak offers a natural context to deeply investigate the determinants of survivor guilt and its association with commonly experienced psychiatric sequelae, with which guilt is known to be associated.

However, some weaknesses must be recognized. First, the limited health care resources prevented us from structurally assessing all the psychopathological features in a single cohort, resulting in a narrowed subsample for the evaluation of cognitive distortion. Secondly, all the participants were in charge of the same service, possibly inducing stratification issues. Lastly, the cross-sectional design of the current study restricts the possibility of accurately outlining the psychopathological drivers and implications of post-COVID survivor guilt. Further investigations on larger samples are required to replicate our findings, possibly taking advantage of longitudinal designs, which may allow for better pinpointing of the affective mechanism underpinning psychiatric sequelae of COVID-19.

Considering the potential role of survivor guilt as a root driver of psychopathology in the post-COVID stages, we encourage psychotherapists to work through guilt feelings in their practice. Moreover, special attention should be given to maladaptive attributional style, which may uphold depressive and post-traumatic symptoms through the exacerbation of survivor guilt emotion. In the face to reshape psychotherapeutic practice in the post-COVID-19 era, clinicians can consider survivor guilt as a core psychopathological issue to tailor specific interventions.

## Figures and Tables

**Table 1:** Descriptive statistics of demographic, clinical, and psychopathological features of the whole sample. Familiar experience of illness: nobody else infected (NEI); somebody infected but not hospitalized (SINH); somebody infected and hospitalized (SIH). Setting of care includes standard care, Non-invasive Ventilation (NIV), and Intensive Care Unit (ICU). Patients self-rated their symptoms on the Impact of Event Scale-Revised (IES-R); Zung Self-Rating Depression Scale (ZSDS); Women’s Health Initiative Insomnia Rating Scale (WHIIRS); IES-ZSDS Discrepancy Score represents a measure to account for the discrepancy in post-traumatic and depressive symptoms severity. Values are presented as means ± SD.

COVID-19 Survivors (n = 195)	
Age	57.29 ± 10.41
Sex (M/F)	132/63
Days after virus clearance	35.82 ± 12.94
Standard Care–NIV (ICU)	147–34 (14)
NEI–SINH (SIH)	50–109 (36)
Survivor Guilt sub-scale	9.02 ± 3.89
WHIIRS	6.87 ± 5.27
IES-R	19.94 ± 18.63
ZSDS index	42.31 ± 10.74
WHIIRS ≥ 9 yes (no)	65 (127)
IES-R ≥ 33 yes (no)	46 (149)
ZSDS index ≥ 50 yes (no)	47 (148)
IES-R–ZSDS Discrepancy Score	−19.65 ± 17.04
Cognition Questionnaire (n = 67)	11.75 ± 7.19

## Data Availability

The data presented in this study are available on reasonable request from the corresponding author.
